# Rapamycin-Resistant mTOR Activity Is Required for Sensory Axon Regeneration Induced by a Conditioning Lesion

**DOI:** 10.1523/ENEURO.0358-16.2016

**Published:** 2017-01-13

**Authors:** Weitao Chen, Na Lu, Yue Ding, Yuan Wang, Leung Ting Chan, Xu Wang, Xin Gao, Songshan Jiang, Kai Liu

**Affiliations:** 1 Division of Life Science, State Key Laboratory of Molecular Neuroscience; 2 Center of Systems Biology and Human Health, The Hong Kong University of Science and Technology, Clear Water Bay, Kowloon, Hong Kong, China; 3 State Key Laboratory of Biocontrol, School of Life Sciences, Sun Yat-sen University, Guangzhou, 510006, China

**Keywords:** axon regeneration, conditioning lesion, dorsal root ganglion, mTOR, spinal cord injury, Stat3

## Abstract

Neuronal mammalian target of rapamycin (mTOR) activity is a critical determinant of the intrinsic regenerative ability of mature neurons in the adult central nervous system (CNS). However, whether its action also applies to peripheral nervous system (PNS) neurons after injury remains elusive. To address this issue unambiguously, we used genetic approaches to determine the role of mTOR signaling in sensory axon regeneration in mice. We showed that deleting mTOR in dorsal root ganglion (DRG) neurons suppressed the axon regeneration induced by conditioning lesions. To establish whether the impact of mTOR on axon regeneration results from functions of mTOR complex 1 (mTORC1) or 2 (mTORC2), two distinct kinase complexes, we ablated either Raptor or Rictor in DRG neurons. We found that suppressing mTORC1 signaling dramatically decreased the conditioning lesion effect. In addition, an injury to the peripheral branch boosts mTOR activity in DRG neurons that cannot be completely inhibited by rapamycin, a widely used mTOR-specific inhibitor. Unexpectedly, examining several conditioning lesion–induced pro-regenerative pathways revealed that Raptor deletion but not rapamycin suppressed Stat3 activity in neurons. Therefore, our results demonstrate that crosstalk between mTOR and Stat3 signaling mediates the conditioning lesion effect and provide genetic evidence that rapamycin-resistant mTOR activity contributes to the intrinsic axon growth capacity in adult sensory neurons after injury.

## Significance Statement

Adult CNS axons usually do not regenerate after injury. Understanding mechanisms of axon regeneration in PNS neurons will help us to develop methods to promote axonal regeneration in the CNS. mTOR is one of the key factors to enhance the regeneration ability of CNS neurons. However, there is no consensus about the effect of mTOR on axon regeneration in the PNS. We provide genetic evidence that mTOR, in particular mTORC1, is required for peripheral lesion–initiated pro-regenerative programs of dorsal root ganglion neurons. Peripheral lesion enhances the rapamycin-insensitive mTOR activity that boosts Stat3 signaling, a transcription factor essential for axon regeneration. Our findings support that injury-induced neuronal mTOR activity in PNS neurons contributes to their intrinsic ability to regenerate axons.

## Introduction

In contrast to the CNS, the adult peripheral nervous system (PNS) axons can spontaneously regenerate after injury. Primary sensory neurons with cell bodies in the dorsal root ganglia (DRG) are widely used as an injury model to study axon regeneration. DRG neurons possess peripheral and central axons. Although peripheral axons can readily regrow after lesion, the ascending axons in the dorsal column of the spinal cord cannot regenerate. This different growth response is due not only to the environment in which the axons are located, but also the growth competence of DRG neurons. It is further illustrated in that a preceding peripheral but not a central branch lesion can accelerate axon regeneration and even promote sensory axons to cross the lesion site after spinal cord injury (McQuarrie and Grafstein, 1973; Richardson and Issa, 1984; Neumann and Woolf, 1999). This conditioning lesion (CL) effect occurs mainly through the regulation of gene expression programs in DRG neurons. Deciphering the mechanism behind the neuronal response specific to the peripheral lesion is important to understand the growth incompetence of CNS neurons.

Ample evidence indicates that peripheral injury signals communicate back to the DRG cell bodies and activate the neuronal genetic program responsible for enhanced axon growth (Rishal and Fainzilber, 2014). Several signaling pathways and transcription factors have been identified to be crucial in the conditioning lesion effect, such as JAK/STAT3, ATF3, cAMP/CREB, Sox11, Smad1, and HIF1a (Qiu et al., 2005; Seijffers et al., 2007; Hannila and Filbin, 2008; Jankowski et al., 2009; Zou et al., 2009; Cho et al., 2015). When activated, some of these transcription factors can also promote the axon regeneration of CNS neurons, consistent with the notion that the axon growth programs can be shared in two types of neurons with different growth competence.

Pten, a negative regulator of PI3K signaling, is one of the critical determinants of intrinsic growth ability in adult CNS (Liu et al., 2011). Pten deletion promotes axon regeneration through both mammalian target of rapamycin (mTOR)–dependent (Park et al., 2008) and –independent (Guo et al., 2016) mechanisms. mTOR signaling integrates nutrients, energy, oxygen, stress, and growth factors to regulate key anabolic and catabolic processes, including protein, lipid, and nucleotide synthesis and autophagy (Laplante and Sabatini, 2012). The role of mTOR signaling in axon regeneration has been investigated in different species, including worm, fly, zebrafish, and rodent (He and Jin, 2016). In rodent retinal ganglion cells (RGCs), the loss of the potential to regrow axons is accompanied by downregulation of mTOR activity in RGCs during maturation, which is further reduced after axotomy. This two-step down-regulation of mTOR activity has been proposed as one of the intrinsic mechanisms by which CNS axons fail to regenerate. In contrast to RGCs, mTOR activity is not downregulated in injured DRG neurons (Abe et al., 2010; Belin et al., 2015), leading to the speculation that it may be linked to neuronal regenerative ability in PNS. However, it has been suggested that mTOR is dispensable for axonal regeneration of adult sensory neurons (Christie et al., 2010; Saijilafu et al., 2013), largely because rapamycin, a specific mTOR inhibitor, does not suppress the axon growth of DRGs. Conversely, mTOR activation through TSC2 deletion increases sensory axon regrowth after peripheral lesion (Abe et al., 2010). Recently, the function of rapamycin-resistant mTOR has been demonstrated in several cell types (Feldman et al., 2009; Thoreen et al., 2009). Therefore, the role of mTOR in the axon regeneration of DRG neurons remains to be elucidated.

mTOR is a serine/threonine kinase that exists in two complexes, mammalian target of rapamycin complex 1 (mTORC1) and 2 (mTORC2), which are differentially regulated and have distinct substrate specificities (Laplante and Sabatini, 2012). In this study, using several floxed mouse strains targeting mTOR, mTORC1, and mTORC2, we provide genetic evidence that mTOR signaling regulates DRG axon regeneration induced by conditioning lesions.

## Materials and Methods

### Animals

All experimental procedures were performed in compliance with animal protocols approved by the Animal and Plant Care Facility at the Hong Kong University of Science and Technology. mTOR^f/f^, Raptor^f/f^ and Rictor^f/f^ mice were obtained from the Jackson Laboratory. Advillin-Cre mice (Hasegawa et al., 2007) were crossed with mTOR^f/f^, Rictor^f/f^ or Raptor^f/f^ mice to produce the conditional knockout mice specifically in sensory neurons. Genotypes were confirmed by PCR at weaning. Both male and female mice were used for experiments. Littermates without Cre were used as controls for all experiments if not mentioned otherwise.

### Surgeries

For all surgical procedures, mice were anesthetized with ketamine (80 mg/kg) and xylazine (10 mg/kg) and received meloxicam (1 mg/kg) as analgesia after the operation.

Sciatic nerve injury was achieved by either transecting or crushing the sciatic nerve. Briefly, the skin and muscle at the middle thigh level was dissected to expose the sciatic nerve. A transection was performed as a conditioning lesion. The nerves on the contralateral side were exposed, but no transection was performed, which acted as a sham control. The sciatic nerve crush injury was conducted using a hemostat (Fine Science Tools) for 30 s. For the CL group, a second crush injury was performed 3 d later proximal to the first crush site.

Dorsal column spinal cord crush was performed using a method similar to previously described methods. Briefly, an incision was made over the thoracic vertebrae, and a laminectomy was conducted to expose the T8 spinal cord. A T8 dorsal hemi-crush injury was performed using modified Dumont #5 forceps at a depth of 0.6 mm. Four weeks later, CTB488 was injected into the sciatic nerve to retrogradely trace the ascending sensory axon. Mice were killed 2 d later for examination. For the acute experiment, CTB was injected shortly after dorsal column crush, and mice were killed after 2 d.

### DRG neuronal culture

DRG primary cultures were performed based on previously described methods. Before tissue harvesting, mice were anesthetized and perfused with sterile PBS. L4, L5, and L6 DRGs were dissected from both the injured side and the contralateral uninjured side as a sham control. The DRGs were placed into HBSS medium containing 0.1% collagenase (Invitrogen). After 90 min of incubation at 37°C, the medium was replaced with 0.25% trypsin for an additional 30 min at 37°C. The trypsin was then inactivated by washing the DRGs with 10% FBS in DMEM. The DRGs were dissociated into single-cell suspensions by triturating in culture medium (Neurobasal, B27, 1% penicillin/streptomycin) 20–30 times. The DRGs were allowed to precipitate at room temperature for 40 min. The supernatants were removed, and the pellets were used for plating. The plates were precoated with poly-d-lysine overnight and laminin (Gibco, 10 μg/mL) for 3–4 h. The cultures were fixed with 4% paraformaldehyde for 15 min. For immunofluorescence staining, the cells were permeabilized and blocked in 4% goat serum and 0.05% Triton X-100 in PBS for 15 min. Staining was performed with the primary antibodies overnight at 4°C and with secondary antibodies for 1 h at room temperature. Images were acquired using a Nikon Eclipse TE2000-E inverted microscope. The average length of the longest axon from neurons was measured and quantified using ImageJ. All measurements were performed in a blinded fashion.

### Immunofluorescence

Primary antibodies against the following proteins were used: GFAP (1:300, Aves Labs), TUJ1 (1:2000, Covance), p-S6 (Cell Signaling Technology), p-mTOR 2448 (1:500, Cell Signaling Technology), p-AKT473 (1:500, Cell Signaling Technology), p-4EBP1 (1:200, Cell Signaling Technology), phospho-Stat3 (1: 300, Cell Signaling Technology), p-cJUN (1:500, Cell Signaling Technology), ATF3 (1:500, Santa Cruz Biotechnology), p-CREB (1:500, Cell Signaling Technology), and SCG10 (1: 2000, Novus). The treated mice were transcardially perfused with PBS followed by 4% paraformaldehyde in PBS. The DRGs, sciatic nerve, brainstem, or spinal cord were dissected and fixed for 12 h in 4% paraformaldehyde in PBS and cryoprotected overnight in 30% sucrose. All samples were embedded in Tissue-Tek O.C.T. and frozen at –80°C. The DRGs, sciatic nerves, brainstem, and spinal cords were sectioned to 8-, 10-, 25-, or 16-μm slices, respectively, using a cryostat and mounted onto coated slides (Thermo Fisher Scientific). The sections were permeabilized and blocked with 4% goat serum and 0.1% Triton X-100 in PBS for 30 min. Primary antibodies were applied to the slides in the blocking solution overnight at room temperature. The primary antibodies were then rinsed with PBS six times in 1 h. Fluorescence-conjugated secondary antibodies (1:500) were incubated for 2 h at room temperature. For p-mTOR 2448, p-AKT473, p-Stat3, p-cJUN, ATF3, p-CREB, and p-4EBP1 staining, heating-mediated antigen retrieval in sodium citrate buffer was conducted before staining.

### Western blotting

Antibodies against the following proteins were used: pS6 ribosomal protein, S6, β-actin, mTOR, Raptor, and Rictor (Cell Signaling Technology). DRGs were placed into lysis buffer with protease inhibitors [50 mM HEPES, pH 7.4, 150 mM NaCl, 10% glycerol, 1.5 mM magnesium chloride, 1 mM EGTA (Sigma-Aldrich), 1 mM sodium vanadate (Sigma-Aldrich), 10 mM sodium pyrophosphate (Sigma-Aldrich), 10 mM NaF (Sigma-Aldrich), 1% Triton X-100, 1% sodium deoxycholate (Sigma-Aldrich), 0.1% SDS, 1 mM PMSF (Sigma-Aldrich), and 1 mM complete mini-protease inhibitor (Roche Diagnostics)]. Tissues were homogenized with a pellet pestle motor (Montes) on ice and centrifuged at 10,000 × *g* for 15 min. The supernatant was taken out, and protein concentration was measured by Bradford reagent assay. The cell lysates were mixed with 5× SDS sample buffer [300 mM Tris-HCl buffer, pH 6.8, 10% (w/v) SDS, 25% (v/v) beta-mercaptoethanol, 50% (v/v) glycerol, and 0.05% (v/v) bromophenol blue] at the ratio of 4:1 and heated at 99°C for 5 min and stored at –80°C. Equal amounts of protein samples were loaded onto SDS gels, and Western blotting procedures were performed according to standard protocols.

### Quantification and statistical analysis

The images were collected under a 10× objective using a confocal microscope (Zeiss, LSM710). For the quantification of p-Stat3, ATF3, p-cJUN, and p-CREB, signal-positive DRG neurons in the nucleus were counted and normalized to the number of total neurons. The number of p-4EBP1–positive neurons in the cytoplasm was counted. Quantification of the sciatic nerve was similar to previously described methods. A column with a width of 50 pixels was drawn at different distances from the lesion center, and the fiber optical density was measured using ImageJ. The distance between the lesion center and the column with half the intensity of the lesion center was considered the regeneration index. Quantification of the ascending sensory axons was based on a previous publication. The axon index was quantified using the number of regenerating fibers at different distances from the lesion center and was normalized to the number of axons at less than –0.4 mm. For all the quantifications, three sections were quantified for each animal to obtain an average number. Student’s *t*-test was used for single comparisons between two groups. The remainder of the data were analyzed using ANOVA. *Post hoc* comparisons were carried out when a main effect showed statistical significance. All analyses were conducted using GraphPad Prism. All bar graphs represent the mean ± SEM.

## Results

### mTOR signaling is required for sensory axon regeneration induced by conditioning lesions

**Figure 1.**
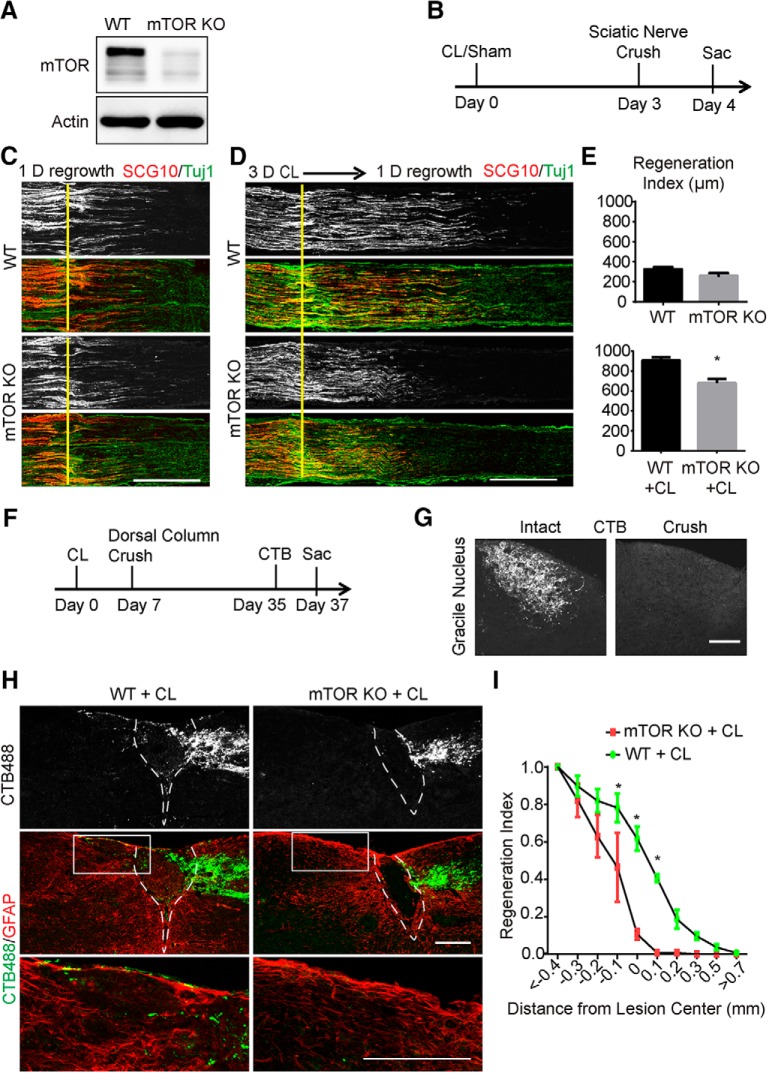
Sensory axon regeneration induced by a conditioning lesion is inhibited by mTOR deletion in DRG neurons. ***A***, DRG lysates were analyzed by Western blot to confirm loss of mTOR in conditional knockout mice. ***B***, Diagram of the experimental procedure assessing the peripheral nerve crush. ***C***, ***D***, Sections of sciatic nerves from WT and mTOR KO mice 1 d after crush, with sham (***C***) or a 3-d CL (***D***). SCG10 (gray and red) staining was used to label the regenerating sensory axons and Tuj1 (green) staining was used to mark the sciatic nerve. Scale bar, 500 μm. ***E***, Quantifications of peripheral axon regeneration. Student’s *t* test. *p < 0.05, four to six mice in each group. ***F***, Diagram of the experimental procedure assessing the ascending sensory axon regeneration after CL. ***G***, Sections of gracile nuclei from mice with or without crush to verify the completeness of the dorsal column crush lesion, with ascending sensory axons traced with CTB (gray). Scale bar, 100 μm. ***H***, Sagittal sections of the spinal cord dorsal columns containing CTB-labeled sensory axons from WT and mTOR KO mice 4 weeks after spinal cord injury. CL was performed 1 week before the spinal cord injury. CTB488 (gray and green) was injected into the sciatic nerve to trace the ascending sensory axons from the L4–6 DRGs. White dashed lines mark the margin of the lesion site indicated by GFAP staining (red). The lower panels show the enlarged boxed area. Scale bar, 200 μm. ***I***, Quantification of traced axons at different distances to the lesion center. Two-way ANOVA followed by Bonferroni multiple comparisons test. *p < 0.005, four to eight mice in each group.

To examine whether mTOR plays a role in the intrinsic axon regenerative capacity of DRG neurons, we used a genetic approach and crossed Advillin-Cre mice (Hasegawa et al., 2007) with mTOR floxed mice (Risson et al., 2009) to generate conditional mTOR knockout mice specifically in sensory neurons (mTOR KO). As expected, Western blot analysis of DRG lysates revealed a considerable reduction of mTOR protein in the mutant compared with the control (Fig. 1*A*). Residual mTOR signal was likely from other nonneuronal cells in DRGs. Then we assessed the peripheral axon regeneration with or without CL (Fig. 1*B*). We crushed the sciatic nerve of WT or mTOR KO mice and allowed the sensory axons to regrow for 1 d before death. SCG10 was used as a marker to specifically label the regenerated sensory axons in the sciatic nerve (Shin et al., 2014). The initial growth of SCG10-positive axons was comparable between the WT and mTOR KO mice, which indicated that mTOR is not required for the early stage of axonal regrowth (Fig. 1*C*, *E*). For the conditioning lesion experiment, we crushed the sciatic nerve, waited for 3 d, and applied a second crush injury proximal to the first crush before allowing the axons to regrow for 1 d (Fig. 1*B*). In the WT mice, axon regeneration was markedly accelerated compared with the mice with a single lesion. However, this effect was suppressed in the absence of mTOR, which demonstrated that mTOR is necessary for the injury-induced acceleration of peripheral axon regeneration (Fig. 1*D*, *E*).

Conditioning lesions promote axon regeneration of not only the peripheral branches but also the central branches after spinal cord injury, where sensory axons encounter an inhibitory environment. We investigated whether blocking mTOR activation could also influence the effect of conditioning lesions on axon regeneration in the dorsal column of the adult spinal cord. One week after a left sciatic nerve lesion, we did dorsal column lesion (DCL) at the T8 spinal cord level, and mice were allowed to recover for 4 weeks after the crush injury. Two days before the mice were killed, all animals received a unilateral injection of the tracer cholera toxin subunit B conjugated with Alexa Fluor 488 (CTB488) to their left sciatic nerve (Fig. 1*F*). We verified that the spinal cord crush lesion was complete based on the absence of CTB488 signal in the brainstem gracile fasciculus (Fig. 1*G*). In WT animals, bundles of CTB488-labeled spinal axons were found in the injured adult spinal cord immediately caudal to the lesion. Some axons were able to grow into the injury site and extended to the rostral spinal cord (Fig. 1*H*). In contrast, in the mTOR KO animals, the majority of the regrown sensory axons were observed in the lesion site, and very few axons crossed the lesion site (Fig. 1*H*). The regenerative index was quantified using the number of regenerating axons at different distances from the lesion center normalized to the number of axons at less than –0.4 mm. There was a significant difference in fluorescence at and rostral to the lesion site between the two groups (Fig. 1*I*). Taken together, our results indicate that mTOR signaling is required for the conditioning lesion effect *in vivo*.

mTOR kinase exists in two complexes with distinct functions, namely, mTORC1 and mTORC2. These complexes functionally depend on their subunits Raptor (mTORC1) or Rictor (mTORC2). To establish whether the impact of mTOR on axon regeneration results from the function of either complex, we conditionally deleted either Raptor or Rictor in DRG neurons by crossing Advillin-Cre mice with Raptor floxed (Raptor KO; Sengupta et al., 2010) and Rictor floxed (Rictor KO; Magee et al., 2012) mice to specifically inactivate mTORC1 or mTORC2 signaling.

**Figure 2.**
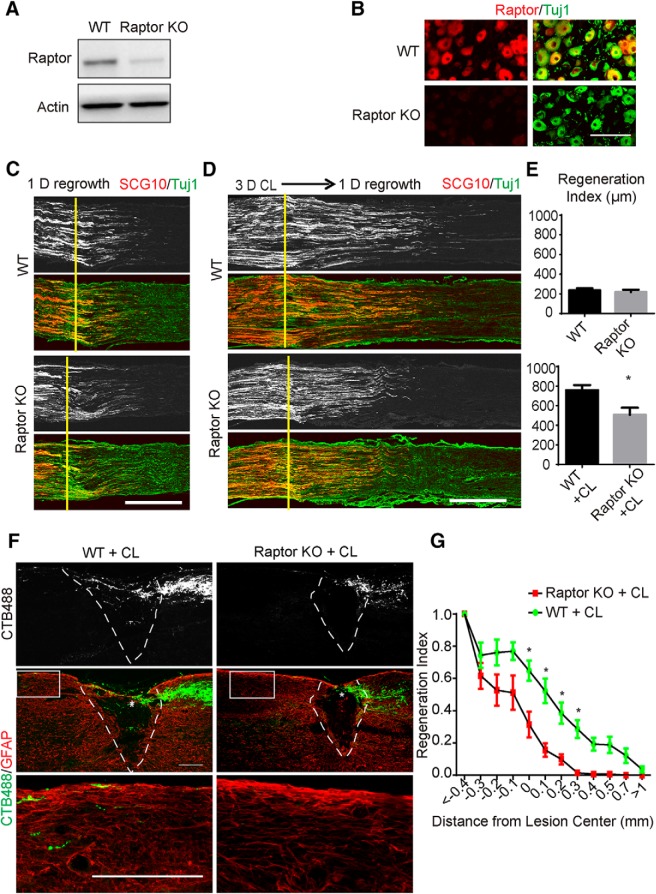
Sensory axon regeneration induced by CL is inhibited by Raptor deletion. ***A***, DRG lysates were analyzed by Western blot to confirm loss of Raptor in the conditional knockout mice. ***B***, DRG sections of WT and Raptor KO mice with immunostaining by Raptor (red) and Tuj1 (green) antibodies. Scale bar, 100 μm. ***C***, ***D***, Sections of sciatic nerves from WT and Raptor KO mice 1 day after crush, with sham (***C***) or 3-d CL (***D***). SCG10 (gray and red) staining was used to label the regenerating sensory axons and Tuj1 (green) staining was used to mark the sciatic nerve. Scale bar, 500 μm. ***E***, Quantifications of peripheral axon regeneration. Student’s *t* test. *p < 0.05, five to seven mice in each group. ***F***, Sagittal sections of spinal cord dorsal columns containing CTB-labeled sensory axons from WT and Raptor KO mice 4 weeks after spinal cord injury. CL was performed 1 week before spinal cord injury. CTB488 (gray and green) was injected into the sciatic nerve to trace the ascending sensory axons from the L4–6 DRGs. White dashed lines mark the margin of the lesion site indicated by GFAP staining (red). The lower panels show the enlarged boxed area. Scale bar, 200 μm. ***G***, Quantification of ascending sensory axon regeneration. Two-way ANOVA followed by Bonferroni multiple comparisons test. *p < 0.005, seven mice in each group.

Raptor deletion was confirmed using Western blotting of DRG lysates (Fig. 2*A*) and immunostaining of DRG sections (Fig. 2*B*). We first examined peripheral regeneration. Raptor knockout did not inhibit the initial regrowth in sensory axons 1 day after sciatic nerve injury (Fig. 2*C*, *E*). In contrast, the acceleration of axon regrowth observed after CL was suppressed with Raptor deletion (Fig. 2*D*, *E*), which was similar to the mTOR knockout mice. We then examined spinal axon regeneration after conditioning lesion. The CL-induced regeneration of the ascending sensory axons was suppressed in the Raptor knockout mice, and very few axons were found beyond the epicenter of the lesion (Fig. 2*F*, *G*). Therefore, mTORC1 signaling is essential for the CL effect in DRG neurons.

**Figure 3.**
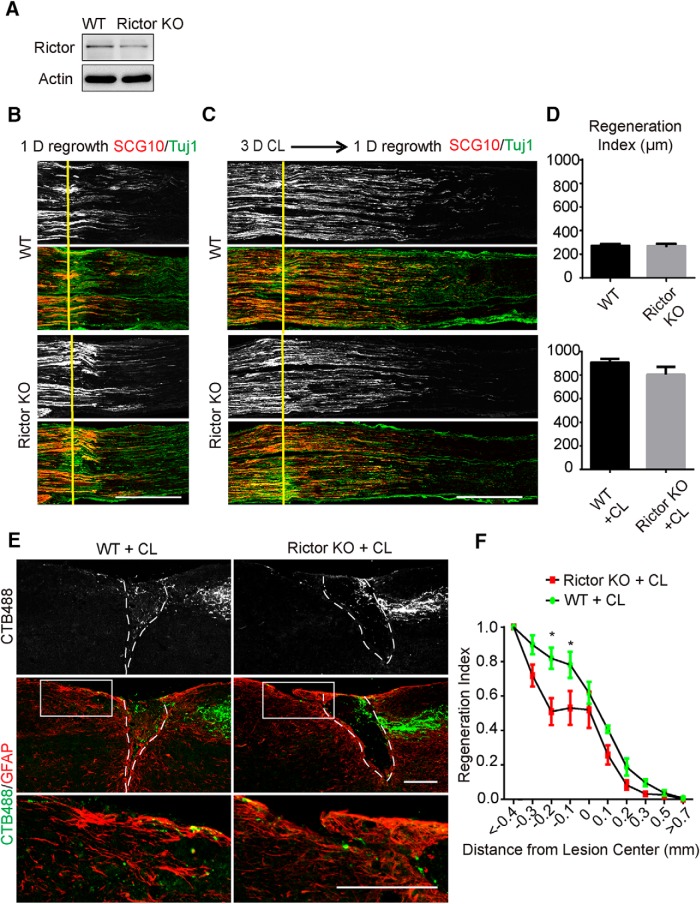
The effect of Rictor deletion on sensory axon regeneration. ***A***, DRG lysates were analyzed by Western blot to confirm loss of Rictor in the conditional knockout mice. ***B***, ***C***, Sections of sciatic nerves from WT and Rictor KO mice 1 d after crush, with sham (***B***) or a 3-d CL (***C***). SCG10 (gray and red) staining was used to label the regenerating sensory axons and Tuj1 (green) staining was used to mark the sciatic nerve. Scale bar, 500 μm. ***D***, Quantifications of peripheral axon regeneration. Student’s *t* test, four to five mice in each group. ***E***, Sagittal sections of spinal cord dorsal columns containing CTB-labeled sensory axons from WT and Rictor KO mice at 4 weeks after spinal cord injury. CL was performed 1 week before spinal cord injury. CTB488 (gray and green) was injected into the sciatic nerve to trace the ascending sensory axons from the L4–6 DRGs. White dashed lines mark the margin of the lesion site indicated by GFAP staining (red). The lower panels show the enlarged boxed area. Scale bar, 200 μm. ***F***, Quantification of ascending sensory axon regeneration. Two-way ANOVA followed by Bonferroni multiple comparisons test. *p < 0.005, seven mice in each group.

Similar experiments were performed in Rictor KO mice. We confirmed Rictor deletion in DRG neurons using Western blotting (Fig. 3*A*). In the sciatic nerve, Rictor knockout had no effect on CL-accelerated sensory axon regeneration (Fig. 3*B*-*D*). In the spinal cord, we observed a modest inhibitory effect on the spinal axon regeneration after dorsal column lesion (Fig. 3*E*, *F*). Therefore, mTORC2 signaling plays a minor role in CL-induced axon regeneration.

**Figure 4.**
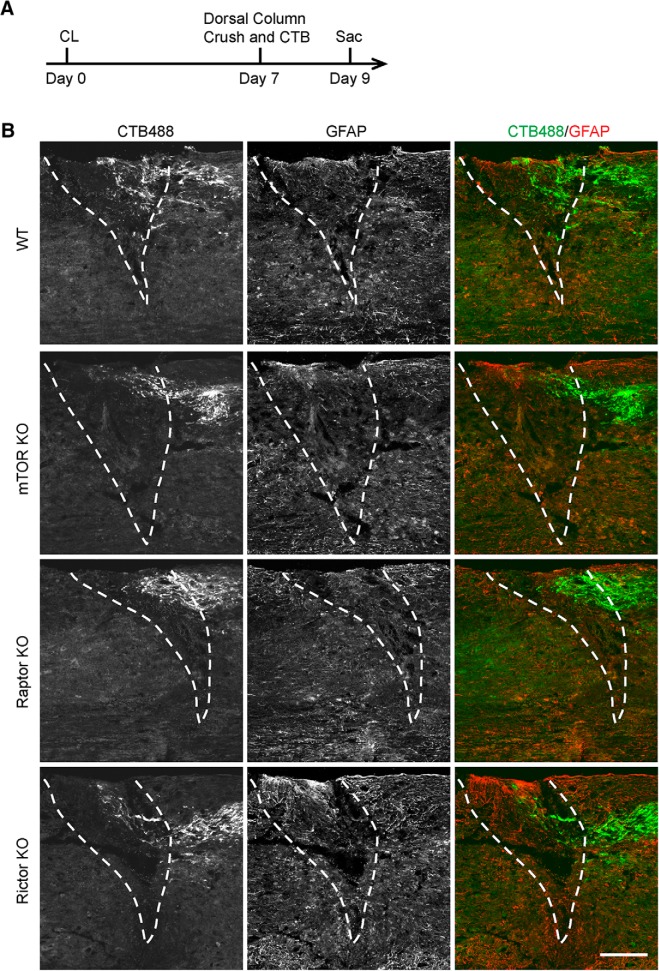
Deletion of mTOR, Raptor, or Rictor does not affect the axon retraction of conditioned sensory axons shortly after dorsal column crush. ***A***, Diagram of the experimental procedure assessing the axon retraction at 2 days after spinal cord injury. ***B***, Representative sagittal sections of spinal cord dorsal columns containing CTB-labeled sensory axons from WT, mTOR, Raptor, and Rictor KO mice at 2 d after spinal cord injury. CL was performed 1 week before the spinal cord injury. CTB488 (green) was injected into the sciatic nerve to trace the ascending sensory axons from the L4–6 DRGs. White dashed lines mark the margin of the lesion site indicated by GFAP staining (red). Scale bar, 200 μm. Note that in all groups, some CTB-labeled sensory axons do not retract from the lesion site.

Conditioning lesions prevent sensory axon dieback after injury (Busch et al., 2009; Ylera et al., 2009). The effects we observed in mTOR or Raptor KO mice could be a result of either axon dieback or suppressed regrowth. Thus, we examined the conditioned sensory axons at 2 d after spinal cord injury (Fig. 4*A*). As previously demonstrated, with a pre–conditioning lesion, some axons in WT mice did not show apparent dieback and located close to the lesion center at 2 d after dorsal column crush (Fig. 4*B*). We observed similar phenotype in mTOR, Raptor, and Rictor KO mice (Fig. 4*B*). Thus, instead of affecting the acute postinjury axonal degeneration, mTOR or Raptor deletion may inhibit the regenerative growth of injured sensory axons with a conditioning lesion.

**Figure 5.**
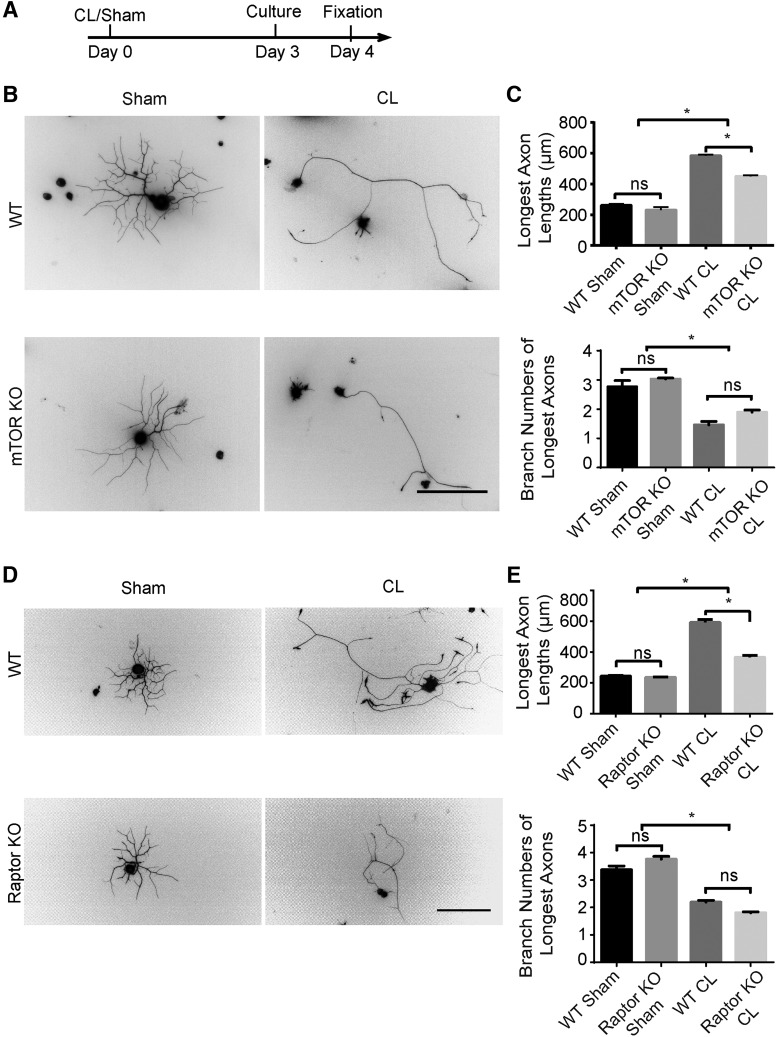
mTORC1 inactivation suppresses the conditioning lesion effect in vitro. ***A***, Diagram of the experimental procedure. ***B***, Primary culture of DRG neurons for 24 h with sham or CL in WT and mTOR KO mice. DRG neurons were stained with Tuj1 antibody. Scale bar, 200 μm. ***C***, Quantification of axon lengths and branch numbers. One-way ANOVA followed by Tukey’s test. *p < 0.05, *n* = 3 mice. ***D***, Primary culture of DRG neurons for 24 h with sham or CL in WT and Raptor KO mice. Scale bar, 200 μm. ***E***, Quantification of axon lengths and branch numbers. One-way ANOVA followed by Tukey’s test. *p < 0.05; ns, not significant; *n* = 3 mice.

To exclude the possibility that the environment such as glial cells or axon degeneration may affect the role of mTOR in CL-induced axon regeneration, we cut the sciatic nerve, waited 3 d, and cultured the isolated adult DRG neurons at low density for 24 h (Fig. 5*A*). We measured the length of the longest axon from each isolated neuron to assess axon elongation. Prior nerve injury markedly potentiated axon growth in the WT DRG cultures (Smith and Skene, 1997). However, this accelerated axonal growth was suppressed in the mTOR KO neurons, but the branch pattern was not changed (Fig. 5*B*, *C*). No significant effect was observed in the neurons on the contralateral side of the mTOR KO mice with sham surgery (Fig. 5*B*, *C*). Elimination of Raptor resulted in a similar downregulation of the CL effect *in vitro* (Fig. 5*D*, *E*). These results demonstrate that mTORC1 is required for the conditioning effect in a cell-autonomous fashion.

### mTOR signaling in the DRG neurons

**Figure 6.**
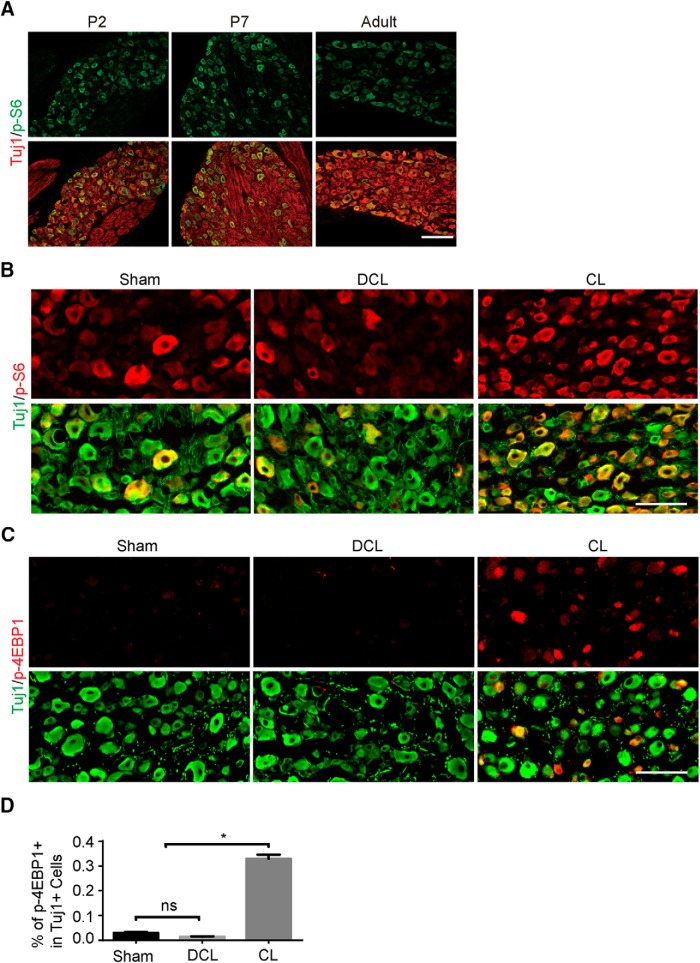
mTOR activity in DRGs during development and after injuries. ***A***, Sections of DRGs from WT mice at postnatal day 2 (P2), P7, and adult, stained with p-S6 (green) and Tuj1 (red) antibodies. Scale bar, 200 μm. ***B***, DRG sections of WT mice with sham surgery, dorsal column lesion (DCL), or peripheral CL, with p-S6 (red) and Tuj1 (green) staining. p-S6 signaling was upregulated 3 d after CL but not DCL. Scale bar, 100 μm. ***C***, DRG sections of WT mice with sham surgery, DCL, or CL, with p-4EBP1 (red) and Tuj1 (green) staining. p-4EBP1 signaling was dramatically upregulated 3 d after CL but not DCL. Scale bar, 100 μm. ***D***, Quantification of percentages of the p-4EBP1^+^ DRGs. One-way ANOVA followed by Tukey’s multiple comparisons test. *p < 0.05; ns, not significant; *n* = 3.

We next evaluated mTOR signaling in the DRG neurons using WT mice. An important function of mTOR is to control protein synthesis through mTORC1. In retinal ganglion cells and corticospinal motor neurons, mTORC1 signaling is downregulated upon maturation and after injury (Park et al., 2008; Liu et al., 2010). In contrast, we found that DRG neurons have similar levels of phosphorylated S6 ribosomal protein (p-S6), an indicator of mTORC1 activity, during development and maturation (Fig. 6*A*). A peripheral nerve lesion enhanced p-S6 (Fig. 6*B*), which is consistent with previous reports. Interestingly, phosphorylated 4EBP1 (p-4EBP1), another downstream target of mTORC1, was barely detectable in naive DRG neurons but was dramatically upregulated by the peripheral lesion (Fig. 6*C*, *D*). In contrast, the dorsal column lesion did not change the level of p-S6 or p-4EBP1 (Fig. 6*B*-*D*). These results suggest that a peripheral rather than central lesion further activates mTORC1 signaling.

**Figure 7.**
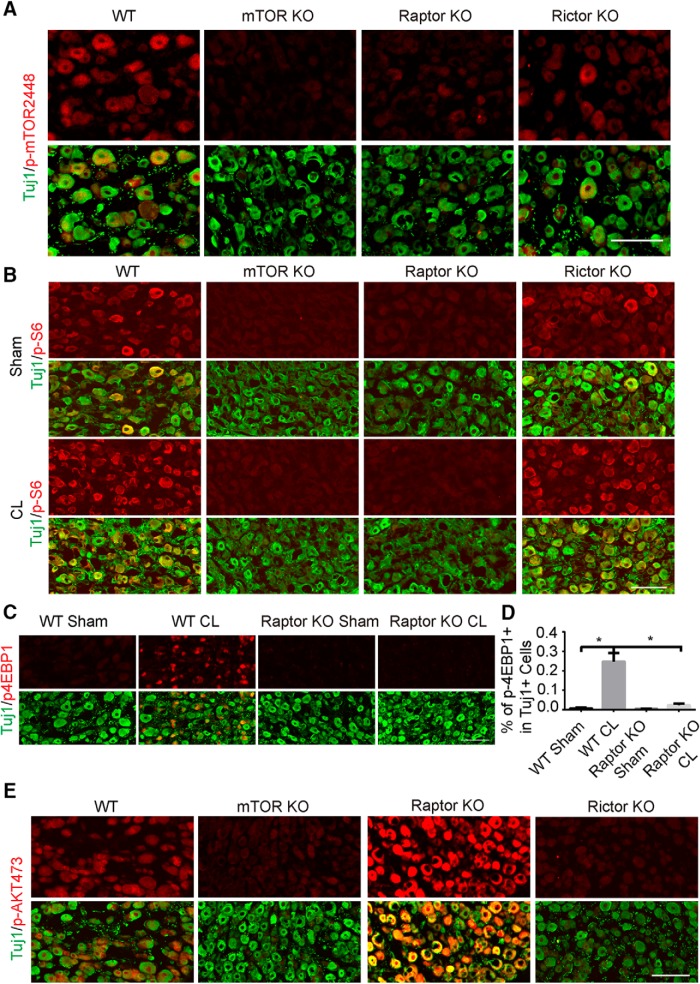
mTOR signaling in DRG neurons of mTOR, Raptor, and Rictor KO mice. ***A***, Sections of DRGs from WT, mTOR, Raptor, and Rictor KO mice, with p-mTOR (red) and Tuj1 (green) staining. Scale bar, 100 μm. ***B***, Sections of DRGs from WT, mTOR, Raptor, and Rictor KO mice with or without CL, with p-mTOR (red) and Tuj1 (green) staining. p-S6 signal was eliminated in mTOR or Raptor KO mice. Scale bar, 100 μm. ***C***, Sections of DRGs from WT and Raptor KO mice with or without CL, with p-4EBP1 (red) and Tuj1 (green) staining. Scale bar, 100 μm. ***D***, Quantification of percentages of p-4EBP1^+^ DRG neurons in three groups of mice. One-way ANOVA followed by Tukey’s test. *p < 0.05, three mice in each group. ***E***, Sections of DRGs from WT, mTOR, Raptor, and Rictor KO mice, with p-AKT473 (red) and Tuj1 (green) staining. Scale bar, 100 μm.

In the DRGs of the mTOR knockout mice, phosphorylated mTOR (p-mTOR) was eliminated (Fig. 7*A*). Similar downregulation of p-mTOR was observed in the Raptor knockout mice, whereas only a modest effect was observed in the Rictor knockout mice (Fig. 7*A*). Ablating mTOR or Raptor but not Rictor blocked the activation of p-S6 in intact and injured DRGs (Fig. 7*B*). Raptor knockout completely blocked the induction of p-4EBP1 by CL (Fig. 7*C, D*). Furthermore, mTOR and Rictor knockout diminished AKT phosphorylation on Ser473 (p-AKT473; Fig. 7*E*), which is an indicator of mTORC2 activation. In contrast, Raptor knockout dramatically elevated the level of pAKT473, suggesting that Raptor may be a negative regulator of mTORC2 (Fig. 7*E*). This finding also suggests that the level of p-AKT473 may not be crucial for the CL effect.

### Rapamycin-resistant mTORC1 facilitates injury-induced Stat3 signaling in DRG neurons

**Figure 8.**
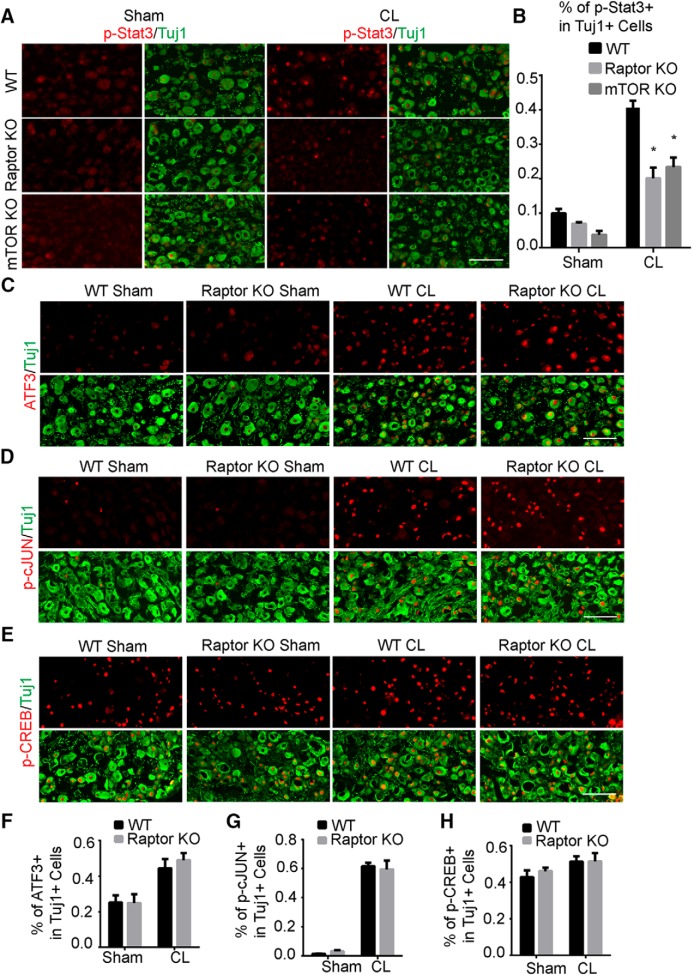
mTORC1 inactivation suppresses Stat3 signaling in DRG neurons induced by a conditioning lesion. ***A***, Sections of DRGs from WT, mTOR, and Raptor KO mice with or without CL, with p-Stat3 (red) and Tuj1 (green) staining. mTOR or Raptor deletion inhibited p-Stat3 at 3 d after CL. ***B***, Quantification of percentages of p-Stat3^+^ DRG neurons in three groups of mice. Two-way ANOVA followed by Tukey’s test. *p < 0.05, three to five mice in each group. ***C***–***E***, Sections of DRGs from WT and Raptor KO mice with or without CL, with ATF3 (***C***), p-cJUN (***D***), and p-CREB (***E***) staining. Tuj1 staining was used to label DRG neurons. ***F***–***H***, Quantifications of percentages of ATF3^+^ (***F***), p-cJUN^+^ (***G***), and p-CREB^+^ (***H***) DRG neurons in three groups of mice. Two-way ANOVA followed by Tukey’s test. *p < 0.05, three to five mice in each group. Scale bar, 100 μm.

We next investigated the mechanism by which mTORC1 upregulation promotes axon regeneration after conditioning lesions. Inactivation of mTOR suppressed the lesion-induced effect but not the initial axon growth, suggesting that a prior injury signal is required. Peripheral CL activates several transcription factors that contribute to axon regeneration. We hypothesized that mTORC1 modulates these injury-induced signals; therefore, we performed immunostaining of several well-established injury-activated pro-regenerative signals, including ATF3, JAK/STAT, cAMP/CREB, and JNK/cJUN pathways, in DRG neurons from WT, mTOR KO, and Raptor KO mice at 3 d after sciatic nerve lesion, when the CL effect on the axon growth was readily detected in culture (Fig. 5). Upon injury, the transcription factor Stat3 was phosphorylated and accumulated in DRG cell bodies. We found that the injury-induced increase in the number of cells expressing p-Stat3 was suppressed in both mutant mice (Fig. 8*A*, *B*). However, the levels of ATF3, p-CREB and p-cJUN were not significantly different between the WT and Raptor KO mice (Fig. 8*C*-*H*), which suggested that the effect was specific for Stat3 rather than a general inhibition of injury signals. It has been shown that Stat3 deletion in DRG neurons dramatically inhibited axon regeneration (Bareyre et al., 2011). Therefore, mTORC1 mediates the full activation of Stat3 signaling in the cell bodies of injured neurons, and its action on conditioning lesions may function, in part, through Stat3. Other downstream signaling of mTORC1 may also contribute to the growth-promoting effect and needs further investigation.

**Figure 9.**
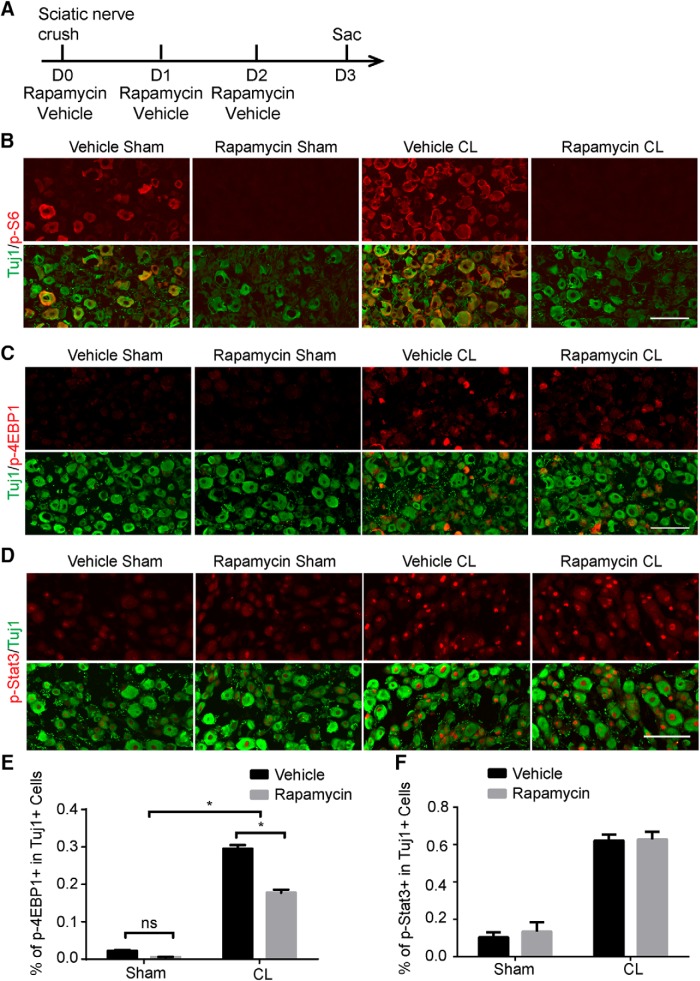
Injury-induced Stat3 signaling is not affected by rapamycin. ***A***, Diagram of the experimental procedure. ***B***–***D***, Sections of DRGs from WT mice with or without CL, with p-S6 (***B***), p-4EBP1 (***C***), or p-Stat3 (***D***) staining (red). Mice were treated with vehicle or rapamycin. Tuj1 staining (green) was used to label DRG neurons. Scale bar, 100 μm. ***E***, ***F***, Quantifications of p-4EBP1^+^ (***E***) or p-Stat3^+^ (***F***) DRG neurons after vehicle or rapamycin treatment. Two-way ANOVA followed by Tukey’s test. *p < 0.05, *n* = 5.

Our experiments demonstrate that mTORC1 is required for the conditioning lesion effect. However, rapamycin, a specific inhibitor of mTOR, is unable to inhibit the regeneration ability of DRG neurons (Christie et al., 2010; Saijilafu et al., 2013). One possibility is that injured DRG neurons may activate mTOR signaling to a stage that cannot be suppressed by rapamycin. To test this hypothesis, we treated WT mice with rapamycin for 3 d (Fig. 9*A*) and found that p-S6 expression was completely blocked in the DRG with or without CL (Fig. 9*B*). However, rapamycin only partially inhibited the induction of p-4EBP1 by CL, which was completely blocked in Raptor KO mice (Fig. 9*C*, *E*). Furthermore, p-Stat3 expression was not affected by rapamycin (Fig. 9*D*, *F*). Overall, our data indicate that peripheral lesions boost rapamycin-resistant mTOR activity, which contributes to the CL lesion effect through the modulation of Stat3 signaling.

## Discussion

In the current study, using conditional knockout mice in DRG neurons, we provide genetic evidence indicating that mTOR and Raptor are required for sensory axon regeneration enhanced by peripheral lesions in mice, whereas Rictor plays a minor role. The peripheral lesion activates rapamycin-resistant mTOR signaling to modulate Stat3 activity and further promotes axon regeneration.

In adult RGCs, mTORC1 activation promotes robust axon regeneration after optic nerve injury. In contrast to CNS neurons, which downregulate mTOR activity after injury, PNS neurons activate mTOR. We showed that removing mTOR eliminates both mTORC1 and mTORC2 activity and dramatically suppresses CL-induced axon regeneration. Elimination of mTORC1 activity through Raptor knockout results in a deficit in axon regeneration that is comparable to mTOR KO. Deletion of Rictor modestly inhibits axon regeneration. In PNS neurons, the deletion of Raptor, but not Rictor, dramatically downregulates mTOR phosphorylation, which indicates a dominant role of mTORC1 in maintaining mTOR activity in DRG neurons. Raptor KO in the DRG neurons enhances AKT473, whereas Rictor KO decreases it, which suggests feedback control of mTORC1 to mTORC2. It has been shown that different isoforms and phosphorylation of AKT play distinguished roles in the retinal axon regeneration (Miao et al., 2016). It will be interesting to examine the precise function of AKT in the DRG axon regeneration.

A peripheral but not a central lesion induces Stat3 activation in DRG neurons within hours, and the signal is sustained for 1–2 weeks. Inhibiting Stat3 either through a chemical inhibitor or genetic deletion dramatically suppresses the axon regeneration of DRGs (Qiu et al., 2005; Bareyre et al., 2011). In RGCs, activation of Stat3 promotes robust axon regeneration in the optic nerve (Sun et al., 2011; Luo et al., 2016). These findings point to the functional significance of this pathway in both CNS and PNS neurons. It has been suggested that Stat3 integrates cytokine and neurotrophin signals to promote peripheral axon regeneration (Pellegrino and Habecker, 2013; Quarta et al., 2014). Multiple studies demonstrated that mTORC1 regulates the activation of Stat3 (Yokogami et al., 2000; Cloetta et al., 2013). However, it is unclear whether mTOR and Stat3 associate with each other to regulate the peripheral axon regeneration. In fact, the Pten/mTOR and JAK-Stat3 pathways function independently to regulate axon regeneration of RGCs in the CNS (Sun et al., 2011). In contrast to RGCs, here we show that in DRGs, the deletion of either mTOR or Raptor inhibits the CL-enhanced expression of p-Stat3. The data suggest the existence of crosstalk between the mTOR and Stat3 pathways. CL-induced Stat3 activity in DRG neurons could be regulated by retrograde mechanisms (Ben-Yaakov et al., 2012) and cytokines (Sendtner et al., 1992). In response to cytokines or growth factors, Stat3 is phosphorylated at Tyr705 by JAK or by receptor tyrosine kinases (RTKs). In addition, STAT3 can also be phosphorylated on Ser727, which is required for the maximal activation (Wen et al., 1995). It has been shown that mTOR could function as one of the kinases that phosphorylate Stat3 at Ser727 (Yokogami et al., 2000; Kim et al., 2009). It is not clear whether mTOR kinase regulates Stat3 activity directly or indirectly in DRG neurons. Our results warrant further study to elucidate the underlining mechanism. In addition, rapamycin cannot inhibit p-Stat3 in DRGs, which is consistent with its ineffectiveness on axon regeneration. Recent systems-level analysis indicates crosstalk among several signaling pathways involved in axonal regeneration after PNS injury (Chandran et al., 2016), supporting the notion that multiple signaling pathways mediate the CL effect.

Protein synthesis is one of the best-characterized functions mediated by mTORC1, although we did not have evidence that mTOR regulates axon regeneration through this function. mTORC1 regulates protein synthesis through its substrates including 4EBP1 and S6K1 (Laplante and Sabatini, 2012). Both 4EBP1 and S6K1 are required for Pten deletion-induced regeneration in RGCs (Yang et al., 2014). The mTOR-dependent regeneration of RGCs is also sensitive to rapamycin. In contrast to RGCs, immunostaining shows that naive adult DRGs express high levels of p-S6, but the level of p-4EBP1 is nearly undetectable. This may suggest a selective maintenance of mTOR activity that is possibly related to metabolic homeostasis (Howell and Manning, 2011). Upon peripheral injury, we speculate that DRG neurons may switch to a different status to enhance protein synthesis under stress by further activating mTORC1 signaling and its substrates, which is evidenced by the marked increase in p-4EBP1 expression. S6K1 does not seem to be crucial for axon regeneration in DRGs, because rapamycin completely suppresses p-S6 but has little effect on axon growth, as previously reported. Raptor KO but not rapamycin completely suppressed p-4EBP1 expression. Numerous functions of mTORC1, including S6K1, are highly sensitive to rapamycin. However, rapamycin-resistant mTOR activity has also been reported. The reasons are not entirely clear. First, as an allosteric inhibitor, rapamycin may partially inhibit protein synthesis depending on the substrates (Kang et al., 2013). In contrast to rapamycin, specific, active-site inhibitors of mTOR that completely inhibit mTORC1 function significantly reduce overall rates of protein synthesis in proliferating cells in vitro (Hsieh et al., 2012). Second, some transcriptional responses are affected only by complete mTOR inhibition (Laplante and Sabatini, 2013). We show that Stat3 activity in DRGs is not suppressed by rapamycin. Third, the presence of feedback loops in the mTOR pathway such as ERK activation may also complicate the effect of rapamycin (Mendoza et al., 2011). An alternative interpretation is that the function downstream of the rapamycin inhibition may be compensated by other signaling pathways in DRGs, such as GSK3β. Nevertheless, our data support a hypothesis that CL may switch on a rapamycin-resistant mTOR activity in DRG neurons. Further understanding the cell context–dependent role of mTOR, especially on injuries, may further illustrate the differential growth ability between CNS and PNS neurons.

## Synthesis

Reviewing Editor: Michael Sofroniew, UCLA School of Medicine Decisions are customarily a result of the Reviewing Editor and the peer reviewers coming together and discussing their recommendations until a consensus is reached. When revisions are invited, a fact-based synthesis statement explaining their decision and outlining what is needed to prepare a revision will be listed below. Note: If this manuscript was transferred from JNeurosci and a decision was made to accept the manuscript without peer review, a brief statement to this effect will instead be what is listed below.

The authors have revised the manuscript in response to previous reviews and have addressed the major concerns.

The findings, although somewhat incremental, convincingly present some new information about interactions between the mTOR and STAT3 signalling pathways in the up regulation of sensory axon regeneration after conditioning lesions.
